# Common electrocardiogram measures are not associated with telomere length

**DOI:** 10.18632/aging.204149

**Published:** 2022-07-05

**Authors:** Aenne S. von Falkenhausen, Rebecca Freudling, Melanie Waldenberger, Christian Gieger, Annette Peters, Martina Müller-Nurasyid, Stefan Kääb, Moritz F. Sinner

**Affiliations:** 1 Department of Cardiology, University Hospital, LMU Munich, Munich, Germany; 2 German Centre for Cardiovascular Research (DZHK), Partner Site Munich Heart Alliance, Munich, Germany; 3 Institute of Epidemiology, Helmholtz Zentrum München, German Research Center for Environmental Health, Neuherberg, Germany; 4 Research Unit Molecular Epidemiology, Helmholtz Zentrum München, German Research Center for Environmental Health, Neuherberg, Germany; 5 Institute for Medical Information Processing, Biometry and Epidemiology, Medical Faculty, LMU Munich, Munich, Germany; 6 Institute of Genetic Epidemiology, Helmholtz Zentrum München, German Research Center for Environmental Health, Neuherberg, Germany; 7 Institute of Medical Biostatistics, Epidemiology and Informatics (IMBEI), University Medical Center, Johannes Gutenberg University, Mainz, Germany; 8 Pettenkofer School of Public Health Munich, Institute for Medical Information Processing, Biometry and Epidemiology, Medical Faculty, LMU Munich, Munich, Germany

**Keywords:** electrocardiogram, telomere length, cardiac aging

## Abstract

Aims: Aging is accompanied by telomere shortening. Increased telomere shortening is considered a marker of premature aging. Cardiac aging results in the development of cardiac pathologies. Electrocardiogram (ECG) measures reflect cardiac excitation, conduction, and repolarization. ECG measures also prolong with aging and are associated with cardiac pathologies including atrial fibrillation. As premature prolongation of ECG measures is observed, we hypothesized that such prolongation may be associated with telomere length.

Methods and Results: We studied the large, community-based KORA F4 Study. Of 3,080 participants enrolled between 2006 and 2007 with detailed information on demographic, anthropometric, clinical, and ECG characteristics, 2,575 presented with available data on leukocyte telomere length. Telomere length was determined by real-time quantitative PCR and expressed relative to a single copy gene. We fitted multivariable adjusted linear regression models to associate the ECG measures RR-interval, PR-interval, QRS-duration, and heart rate corrected QTc with telomere length.

In our cohort, the mean age was 54.9±12.9 years and 46.6% were men. Increased age was associated with shorter telomere length (p<0.01), and men had shorter telomere length than women (p<0.05). In unadjusted models, heart rate (p=0.023), PR-interval (p<0.01), and QTc-interval (p<0.01) were significantly associated with shorter telomere length. However, no significant associations remained after accounting for age, sex, and covariates.

Conclusions: ECG measures are age-dependent, but not associated with shortened telomere length as a marker of biological aging. Further research is warranted to clarify if shortened telomeres are associated with clinical cardiac pathologies including atrial fibrillation.

## INTRODUCTION

Electrocardiogram (ECG) recordings are widely used in clinical routine. Quantitative ECG measures thereby reflect cardiac excitation (RR-interval), atrial (PR-interval) and ventricular (QRS-duration) conduction, and cardiac repolarization (heart rate corrected QTc). All mentioned ECG measures are age-dependent [1]. Some individuals present with altered ECG measures, for example with early-onset PR prolongation, before reaching an expected calendar age. It is unclear if such premature changes reflect underlying subclinical pathologies, or if these individuals are affected by a premature biological age, which does not correspond with their calendar age.

Telomere length has gained scientific interest as a marker of biological aging [2]. Telomeres are tandem repeats of six nucleotides (TTAGGG) located at the end of each chromosome. Intact telomeres prevent spontaneous DNA damage and preserve genomic integrity. However, telomeres shorten during each mitotic cell cycle. As cellular lifetime progresses, telomere shortening leads to apoptosis [3, 4]. Men have shorter telomeres on average compared to women [5]. Furthermore, telomere shortening has been linked to numerous common conditions including obesity, smoking, hypertension, elevated plasma cholesterol levels, cancer, and cardiovascular diseases [6–13].

Here we used data from the large and well-characterized, community-based KORA Study to systematically test associations between ECG measures and telomere length. We hypothesized that shorter telomeres reflect advanced biological age and are associated with age-dependent changes in ECG measures.

## MATERIALS AND METHODS

### Study population

The community-based KORA (Cooperative Health Research in the Region of Augsburg) Study has been conducted since 1984 in the population living in and around Augsburg, Germany [14]. From 1999 to 2001, 4,261 individuals age 25-74 years of German nationality, randomly selected through the registration office, were enrolled into the KORA S4 survey. The KORA F4 survey was performed as a seven-year follow-up of KORA S4, conducted between 2006 and 2007. Overall, 3,080 individuals (79.6% of KORA S4) agreed to participate. Details have been reported elsewhere [15]. All participants provided written informed consent to participation in the study. The investigations were carried out in accordance with the Declaration of Helsinki. All study methods were approved by the ethics committee of the Bavarian Chamber of Physicians, Munich (S4: EC No. 99186 and for genetic epidemiological questions 05004, F4: EC No. 06068).

### Clinical covariates and electrocardiogram recording

**Figure 1:**
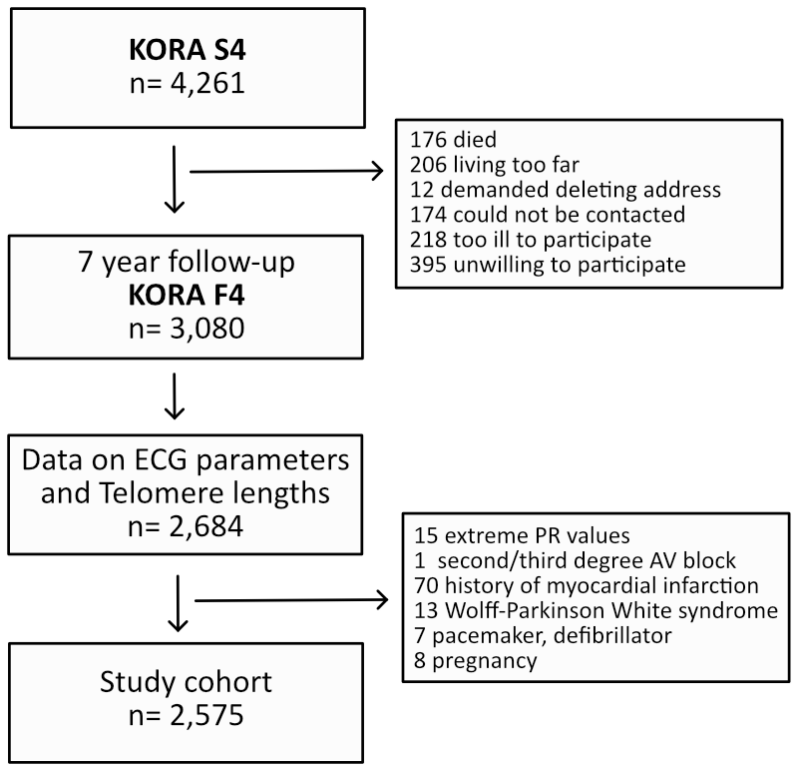
Illustration of the cohort composition.

All participants received an assessment of demographic, anthropometric, and clinical characteristics through a standardized personal interview, a physical examination, and a self-administrated questionnaire. All participants further received a twelve-lead Electrocardiogram (Hörmann Bioset 9000) recorded under standardized conditions after 10 min rest in supine position. For the present study, ECGs were filtered for ECG-quality and analyzed using the automated Hannover ECG System (HES) as reported before [16, 17]. We excluded participants presenting with extreme PR values (≤80ms or ≥320ms), second or third degree atrioventricular block, atrial fibrillation, a history of myocardial infarction, Wolff-Parkinson-White syndrome, or in the presence of a pacemaker or implantable cardioverter defibrillator. We further excluded currently pregnant women (Figure 1). For the presented analyses we tested the four commonly used ECG parameters, RR-interval, PR-interval, QRS duration, and QTc corrected using Bazett’s formula.

### Measurement of telomere length

Participants provided biosamples for laboratory assessment including genetic analyses upon enrollment into the community-based KORA study and at the same visit as all other measures and characteristics. In all those with available peripheral blood biospecimens, we determined telomere length as previously described in detail [18]. In brief, DNA was extracted from peripheral blood leucocytes and telomere length was determined using a quantitative PCR-based technique. By expressing telomere length as the T/S ratio of the telomere repeat copy number (T) to the single copy gene 36B4 (S), we standardized results irrespective of PCR cycles. For quality control, a standard DNA from a K562 cell line was used to assess variation across PCR plates, and duplicates were used to assess intra-sample variability. The coefficient of variation of telomere length was 3.1%.

### Statistical analysis

Discrete data are presented as absolute and relative frequencies. Continuous variables are shown as mean ± standard deviation. Telomere length is used as a continuous variable and is considered normally distributed. Telomere length is compared by sex using the Welch two sample t-test. An unadjusted linear regression is fitted for telomere length and age. We then fitted multivariable-adjusted linear regression models to associate the outcome of ECG measures with the predictor telomere length. We accounted for age, sex, height, and body-mass-index. We present sex-stratified results. All statistical analyses were performed using Rstudio (Version 1.2.1335, Boston, MA, USA). Significance was assumed for a two-sided p <0.05.

## RESULTS

The study flow is visualized in Figure 1. Of 3,080 participants enrolled in the KORA F4 study, 2,684 had complete availability of ECG and telomere length data. Of these, 109 individuals fulfilled any exclusion criteria. Hence, 2,575 individuals were included into the final analysis. Baseline characteristics of the study cohort and the distribution of their ECG measures are listed in Table 1. The cohort’s mean age was 54.9±12.9 years and 53.4% were females.

**Table 1:** Cohort characteristics.

	**n=2,575**
**Demographics**	
Age; years	54.9±12.9
Male sex; n (%)	1,200 (46.6%)
**ECG measures**	
RR-interval; ms	944±144
PR-interval; ms	167±23
QRS-duration; ms	92±9
QTc-interval; ms	425±20
**Telomere assessment**	
Relative telomere length	1.86±0.33

Telomere length expressed relatively using a T/S ratio of measured telomere length (T) divided by the copy number of the single copy gene 36B4 (S).

**Figure 2:**
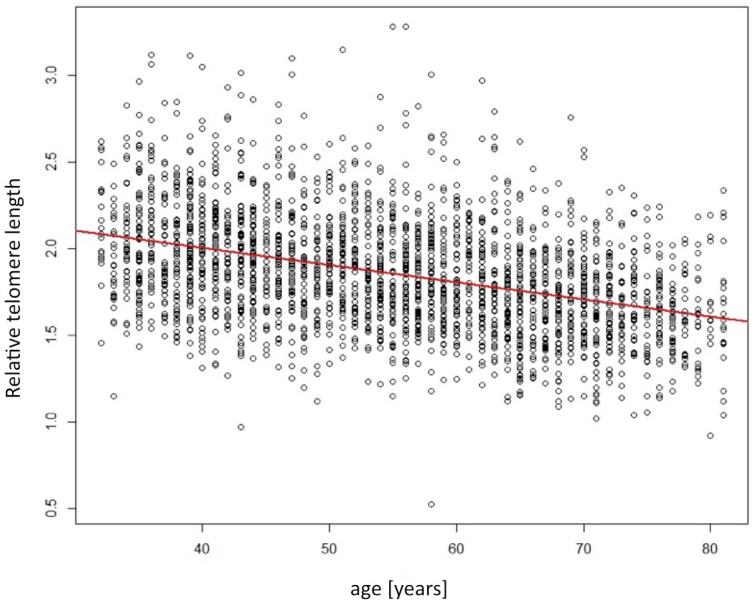
**Scatterplot showing the relation of telomere length (y-axis) depending on age (x-axis).** Red line indicates the linear regression line.

The mean telomere length, expressed as the T/S ratio relative to the single copy gene 36B4, was 1.86±0.33. As previously reported, men had significantly shorter telomeres compared to women (1.81 vs. 1.90, p<0.001). Also, telomeres were significantly shorter in older individuals. The age-dependent relation is depicted in Figure 2. Per year of age, the decrease was -0.0099 relative T/S units (standard error 0.0005; p<0.001). This age-dependent relation was likewise observed when stratified by sex (data not presented).

The associations between ECG measures and telomere length are summarized in Table 2. By unadjusted regression, we found a significant relation between telomere length and RR-interval (p=0.023), PR-interval (p<0.001), and QTc (p<0.001). Regarding the directionality of effects, a shorter telomere length associated with a shorter RR-interval, i.e., a higher heart rate, but inversely correlated with a longer PR-interval and a longer QTc, respectively. However, after accounting for age and other covariates, no significant association between ECG measures and telomere length remained. Also, sex-stratified, adjusted analyses revealed no significant associations (Table 2).

**Table 2:** Regression results.

	**Model 1**		**Model 2**	
	**Beta (SE)**	**p**	**Beta (SE)**	**p**
**RR-interval**	3.1x10^-5^ (4.2x10^-5^)	0.46		
Men			3.7x10^-5^ (5.8x10^-5^)	0.52
Women			2.0x10^-5^ (6.2x10^-5^)	0.75
**PR-interval**	1.5x10^-4^ (2.7x10^-4^)	0.58		
Men			-1.2x10^-4^ (3.7x10^-4^)	0.74
Women			4.6x10^-4^ (3.8x10^-4^)	0.23
**QRS-duration**	7.3x10^-4^ (7.2x10^-4^)	0.31		
Men			6.0x10^-4^ (1.0x10^-3^)	0.56
Women			7.8x10^-4^ (1.0x10^-3^)	0.43
**QTc-interval**	-8.6x10^-3^ (9.9x10^-3^)	0.38		
Men			-6.2x10^-3^ (1.4x10^-2^)	0.66
Women			-9.6x10^-3^ (1.4x10^-2^)	0.49

The table lists the beta coefficients (Beta) and standard errors (SE) of linear regression models for the ECG measures as outcomes and telomere length as predictor. Unadjusted models are described in the manuscript text only. Model 1 is adjusted for age, sex, height, and body-mass-index. Model 2 presents sex-stratified results adjusted for age, height, and body-mass-index.

## DISCUSSION

In the large, community-based KORA study, we associated commonly determined ECG measures with peripheral blood leukocyte telomere length as a marker of biological aging. Despite a known age-dependence of the ECG measures, we found no effect of telomere length beyond the influence of calendar age.

The ECG measures in our study reflect the full cardiac cycle from excitation (RR) via atrial (PR) and ventricular (QRS) conduction to ventricular repolarization (QTc). All measures thereby can be influenced by external factors. Such external conditions exemplarily include heart failure and myocardial infarction, which may result in QRS-prolongation [19]. Various drugs can prolong both the PR-interval and QTc [20, 21]. Importantly, also advancing age is an external factor influencing these ECG measures [1]. Consequently, elderly individuals often require a pacemaker due to a symptomatic slowing of heart rate or a symptomatic atrio-ventricular conduction block [22].

Since aging per se is a non-avertible process, the question arises if a biological age differing from the person’s calendar age is a marker that can be approximated clinically. Telomere length is a well-established marker of biological age [2]. For various clinically relevant cardiovascular conditions, an association with telomere length beyond the effect of calendar age has been shown [2, 11].

With the results of our presented analysis, we confirm the prior notion that predominantly RR-interval, PR-interval, and QTc are indeed age-related [23–26]. All three measures show a highly significant association with telomere length as a marker of age in general. However, after accounting for the effect of calendar age, no significant association with telomere length remained. We therefore conclude that biological age, as measured by telomere length, beyond the effect of calendar age is not a relevant contributor to changes in the investigated ECG measures in a community-based cohort.

Whereas telomere length has been studied for the relation to various clinical conditions and markers [6–10], only limited data exist regarding their role on ECG measures. The available prior data are conflicting. A small study in 273 Australian patients with or without diabetes mellitus described a weak correlation with QRS-duration depending on telomere length status [27]. Another study in 222 Japanese patients presenting with several predominantly chronic neurologic conditions investigated ECG measures in relation to semi-quantitatively measured telomere length using Southern blot analysis. The authors report an association with ECG measures including PR-interval, QRS-duration, and QTc [28]. Importantly, both studies did not adjust for age. To the best of our knowledge, the only prior study that investigated ECG measures in relation to telomere length and did account for calendar age is a small investigation in 139 healthy Chinese patients. Telomere length was semi-quantitatively determined using restriction fragment length analysis and the authors did not find a correlation with ECG measures after age adjustment [29]. Given the limitations of the existing data, we present a most systematic analysis of ECG measures representing the full cardiac cycle. Further, we study a well characterized cohort that represents the German general population and is not restricted to a specific underlying disease phenotype. Most importantly, our analyzed cohort comprises 2,575 individuals. It is hence almost ten-fold larger than prior reports. This cohort size warrants sufficient statistical power to also detect weak association signals. We are thus confident that indeed no relevant effect of telomere length on ECG measures is missed due to a lack of power.

Yet, some considerations are required when interpreting our findings: The KORA study enrolled participants of European descent. Since telomere length shares a heritable component [30], our results may not be fully generalizable. Also, we studied a community-based sample where possible pre-existing diseases and conditions have a low prevalence. It may thus be that our findings cannot be extrapolated to large cohorts of patients presenting with a specific underlying condition. We assessed telomere length in peripheral blood leucocytes, where results do not necessarily reflect fully the telomere length in cardiac tissue. Yet, prior data suggest a high correlation across tissues [31]. Furthermore, we were not able to investigate different leukocyte subtypes, which may be characterized by differential telomere length results that may hence have influenced the association with ECG measures. Most importantly, prolongation of the studied ECG measures can itself predispose to clinical conditions. Exemplarily, PR-interval prolongation is an established risk factor for atrial fibrillation (AF) [32]. Even though we did not find a relevant association between ECG measures and telomere length, it remains unresolved if telomere length is relevant for the pathophysiology of selected age-dependent conditions. At least for AF, a relation with telomere length has been suggested, but the results remain conflicting [33–35].

In conclusion, ECG measures are clearly age-dependent. However, in a large, well-characterized, and sufficiently powered cohort we were not able to substantiate the hypothesis that telomere length as a marker of biological age is a relevant contributor to this age-dependent prolongation of ECG measures. It remains to be resolved if telomere length is involved in the pathophysiology of cardiac diseases like AF that are correlated with a prolongation of ECG measures.
